# Efficacy of Facial Botulinum Toxin A Injections in Alleviating Neuropsychiatric Symptoms in Parkinson's Disease Patients: An Open‐Label, Nonrandomized Controlled Trial

**DOI:** 10.1002/brb3.70806

**Published:** 2025-09-08

**Authors:** Huimin Li, Ming Wei, Xiaofeng Zhu, Xiu Yang, Qiang Tong, Qiu Han

**Affiliations:** ^1^ Department of Neurology Huai'an First People's Hospital, the Affiliated Huai'an No. 1 People's Hospital of Nanjing Medical University Huai'an Jiangsu China

**Keywords:** botulinum toxin A, Cornell Medical Index, neuropsychiatric symptoms, Parkinson's disease

## Abstract

**Background and purpose:**

Parkinson's disease (PD), a prevalent neurodegenerative disorder characterized by motor impairments, frequently accompanied by neuropsychiatric symptoms that significantly impair daily functioning and quality of life. The present study aimed to assess the efficacy of botulinum toxin A (BTX‐A) in alleviating neuropsychiatric symptoms among PD patients.

**Methods:**

This is an open‐label, nonrandomized controlled trial. The subscales of the Cornell Medical Index, including somatization, anxiety, depression, maladjustment, sensitivity, anger, and tension, were used to evaluate neuropsychiatric symptoms. The study compared neuropsychiatric health status among 97 PD patients with neuropsychiatric symptoms, divided into two groups: BTX‐A (*n* = 58) and citalopram hydrobromide (CH; *n* = 39). The BTX‐A group received local injections targeting the bilateral glabella, orbicularis oculi muscle, forehead, bilateral lateral canthus, and temporal area. Patients in the CH group received daily doses ranging from 10 to 40 mg. The efficacy of BTX‐A was assessed before and eight weeks after treatment, with outcomes compared to those of the CH group.

**Results:**

The BTX‐A group showed significant reductions in somatization (*p* = 0.028), tension (*p* < 0.001), anxiety (*p* = 0.001), depression (*p* = 0.002), sensitivity (*p* = 0.006), and total scores (*p* = 0.009) at 8 weeks after treatment. Both the BTX‐A and CH groups demonstrated significant reductions in neuropsychiatric symptoms, with BTX‐A showing comparable efficacy to CH (*p* > 0.05 for all parameters). Additionally, both groups showed comparable neuropsychiatric symptom improvement rates (64.3% vs. 73.7%, *p* > 0.05). These rates were calculated based on reductions in overall neuropsychiatric symptom severity scores.

**Conclusions:**

In summary, BTX‐A demonstrates efficacy in reducing multiple neuropsychiatric symptoms (such as tension, anxiety, depression, sensitivity, etc.) in PD, with comparable effectiveness to CH, supporting its consideration as an alternative therapeutic option.

## Graphical abstract

‐ Group 1: BTX ‐A (n = 58) → Local injections → 8 ‐week follow ‐up.
‐ Group 2: CH (n = 39) → Daily doses (10 ‐40 mg) → 8 ‐week follow ‐up.

Key findings:

‐ Significant reduction in neuropsychiatric symptoms in both groups (P<0.05).
‐ Comparable efficacy between BTX ‐A and CH (P>0.05).
‐ Similar improvement percentages (64.3% vs. 73.7%, P>0.05).

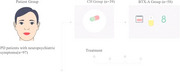

## 1 Introduction

Parkinson's disease (PD), a neurodegenerative disorder characterized by motor impairments, also significantly impacts patients' quality of life through neuropsychiatric symptoms such as depression and anxiety (Bai et al. 2022; Bloem et al. 2021; Leite Silva et al. 2023; Armstrong and Okun 2020). In addition to causing physical discomfort, PD also has a substantial impact on patients' social functioning and neuropsychiatric health  (Aarsland and Kramberger 2015; Cong et al. 2022; de la Riva et al. 2014; Weintraub, Aarsland, Biundo et al. 2022, Weintraub et al. 2015). While oral antidepressants are commonly used, their side effects and compliance issues limit their effectiveness. Emerging evidence suggests botulinum toxin A (BTX‐A) may offer a viable alternative. This study aims to evaluate the efficacy of BTX‐A in managing neuropsychiatric symptoms in PD patients supporting Table.

Several randomized controlled trials have demonstrated the efficacy of BTX‐A in alleviating depressive symptoms, highlighting its potential as a safe and well‐tolerated alternative to conventional antidepressants (Magid et al. 2014; Finzi and Rosenthal 2014; Wollmer et al. 2012; Wang et al. 2022; Dong et al. 2019; Zamanian et al. 2017; Brin et al. 2020; Li et al. 2022; Zhu et al. 2021); Zhu et al. (2021) demonstrated the efficacy of BTX‐A in treating PD and comorbid depression, further supporting its therapeutic potential. This investigation revealed comparable therapeutic efficacy between BTX‐A and sertraline (a first‐line antidepressant) in managing comorbid depression among PD patients, with BTX‐A showing superior safety via fewer adverse events. Although BTX‐A's therapeutic effects have been explored in primary depression and other conditions, its application in managing neuropsychiatric symptoms in PD remains underexplored. Given its favorable safety profile and ease of administration, BTX‐A may represent a promising treatment modality for PD patients experiencing depression and anxiety. Despite evidence supporting BTX‐A's role in treating depressive symptoms, its efficacy in PD‐associated neuropsychiatric manifestations has not been systematically evaluated. This study seeks to fill this gap by comparing BTX‐A with citalopram hydrobromide (CH), a widely used antidepressant.

Emerging evidence suggests progressive escalation in the prevalence and clinical severity of neuropsychiatric comorbidities among PD populations, with heterogeneous pathophysiological mechanisms underlying both isolated and overlapping symptom clusters (Weintraub, Aarsland, Chaudhuri et al. 2022; Ffytche et al. 2017). Clinical observations indicate that conventional pharmacotherapies for these manifestations demonstrate variable therapeutic responses, necessitating chronic administration with attendant risks of polypharmacy complications and suboptimal treatment adherence. In contrast, BTX‐A offers sustained efficacy, favorable safety (localized reactions only), no dopaminergic interactions, and lower annual costs versus oral agents, collectively improving treatment sustainability and quality of life (Lyu et al. 2019). Consequently, local administration of BTX‐A has emerged as a viable treatment option for these patients. However, there is currently a paucity of reports on the ability of BTX‐A to ameliorate neuropsychiatric symptoms in PD patients. This study aims to evaluate the efficacy of local BTX‐A injections in alleviating neuropsychiatric symptoms in PD patients, hypothesizing that BTX‐A will provide comparable improvements to CH with a superior safety profile.

## 2 Methods

### 2.1 Study Design

This trial received ethical clearance from the institutional review board of Huai'an First People's Hospital (approval no. JS‐2024‐150‐01), conducted in full compliance with the Declaration of Helsinki. Designed as a single‐site, nonrandomized, open‐label comparative study between October 2019 and February 2024, the protocol enrolled 189 consecutive PD patients from the neurology outpatient and inpatient departments following informed consent.

### 2.2 Participants

**FIGURE 1:**
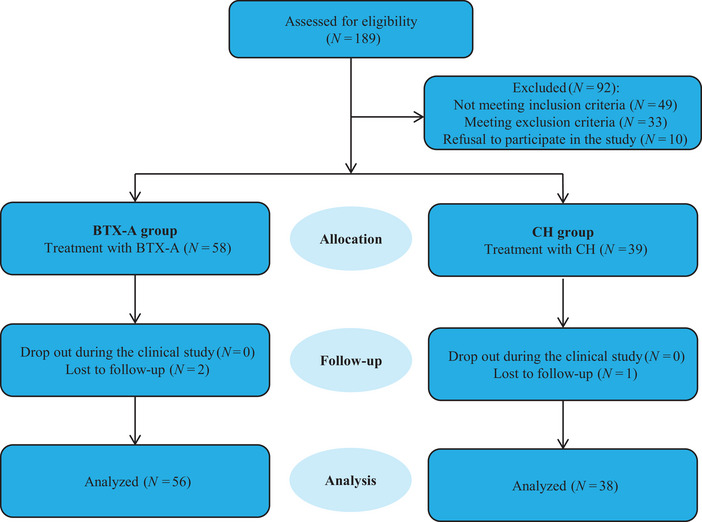
Flowchart of the study.

Diagnostic confirmation of PD was established following the UK Parkinson's Disease Brain Bank diagnostic criteria (Reichmann 2010). The spectrum of neuropsychiatric manifestations in PD represents a common comorbidity, encompassing affective disturbances, behavioral alterations, and psychotic phenomena (Weintraub et al. 2022). Neuropsychiatric comorbidities were assessed using validated scales, yielding 97 participants with measurable affective/psychotic symptoms. These individuals were allocated to either BTX‐A injection therapy (*n* = 58) or oral CH (10–40 mg/day; *n* = 39) after providing written informed consent or through legal guardianship. Post‐allocation attrition resulted in final cohorts of 56 (BTX‐A) and 38 (CH) participants (Figure 1). All self‐reported assessments were completed voluntarily, with proxy responses permitted for participants with severe communication deficits.

### 2.3 Inclusion Criteria

1. Conformity to established diagnostic standards for idiopathic PD per UK Brain Bank criteria.
2. Documentation of ≥2 clinically significant abnormalities on standardized neuropsychiatric assessment instruments.
3. Independent validation of psychiatric comorbidities by two experienced psychiatrists.

### 2.4 Exclusion Criteria

1. Age ≥ 91 years at enrollment.
2. Patients with familial or genetic forms of PD, or those diagnosed with early‐onset PD (defined as onset before 50 years of age).
3. Hoehn–Yahr (HY) stage IV‐V disease severity, indicating advanced motor complications and functional dependency.
4. Clinically significant cognitive deficits compromising assessment validity.
5. History of major psychiatric illness (schizophrenia spectrum disorders, bipolar I disorder, recurrent major depression) or first‐degree relatives with Diagnostic and Statistical Manual of Mental Disorders 5th ed. (DSM‐5) psychotic disorders.
6. Current/recent (≤3 months) exposure to antipsychotic agents, mood stabilizers, or psychotropic medications with Central Nervous System (CNS) activity.
7. Documented hypersensitivity to botulinum neurotoxin formulations or albumin components.
8. Medically unstable conditions (e.g., New York Heart Association (NYHA) Class III‐IV heart failure, end‐stage renal disease, metastatic malignancy) or life expectancy < 12 months.

### 2.5 Cornell Medical Index Self‐Assessment

Before and 8 weeks after treatment, all enrolled participants completed the Cornell Medical Index (CMI) self‐report inventory (Brown and Fry 1962) to evaluate multidimensional health parameters, including somatization tendencies, comprehensive medical history, functional health status, fatigue severity, lifestyle habits, and psychological domains (depression, anxiety, maladaptive behaviors, sensory hypersensitivity, anger expression, and generalized tension). Intergroup comparative analyses were conducted to evaluate statistical differences in these parameters between the BTX‐A group and the CH intervention arm. Furthermore, participants in the BTX‐A group completed a posttreatment CMI reassessment, enabling longitudinal analysis of symptom trajectories relative to their preintervention baselines.

### 2.6 Other Neuropsychiatric Scales

Serving as the primary psychometric tool, the Symptom Checklist‐90 (SCL‐90) quantified psychopathological burden in PD cohorts through standardized self‐assessment. This 90‐item inventory systematically evaluates nine interrelated domains of mental health: anxiety, depressive symptoms, somatoform complaints, cognitive rigidity, interpersonal distress, anger dysregulation, phobic avoidance, suspicious ideation, and reality distortion. By generating dimensional profiles across these domains, the SCL‐90 enabled comprehensive characterization of participants' neuropsychiatric status. To enhance diagnostic precision for affective disorders, the Hamilton Anxiety Rating Scale (HAMA) and Hamilton Depression Rating Scale (HAMD) were incorporated as ancillary measures. Recognized as clinical benchmarks, the HAMA provides granular quantification of anxiety severity through 14 items addressing psychic (e.g., worry, tension) and somatic (e.g., cardiac, gastrointestinal) manifestations, while the 21‐item HAMD delineates depressive symptom clusters including affective disturbance, guilt, suicidality, and neurovegetative features. This multimethodological approach facilitated cross‐validation of subjective experience (self‐report) and objective symptom severity (clinician‐rated scales), thereby strengthening the diagnostic framework.

### 2.7 UPDRS Motor Scores and Hoehn–Yahr Stage

The motor component of the Unified Parkinson's Disease Rating Scale (UPDRS) served as the primary outcome measure for quantifying motor symptom severity in the study (Movement Disorder Society Task Force on Rating Scales for Parkinson's Disease 2003). As a cornerstone clinical assessment tool, the HY staging system provides categorical evaluation of disease progression, delineating five functional stages that reflect evolving motor disability and therapeutic response patterns (Hoehn and Yahr 1967). This ordinal classification system offers critical prognostic value by characterizing disease severity through objective milestones (e.g., unilateral involvement, bilateral impairment, postural instability). Furthermore, the UPDRS enables granular quantification of specific motor domains through standardized clinical examinations, including resting tremor amplitude, lead‐pipe rigidity, bradykinetic task performance, and axial motor control. These evaluations provide objective benchmarks for tracking longitudinal symptom progression and treatment efficacy in clinical practice and research settings.

### 2.8 Local Injection of BTX‐A

**FIGURE 2:**
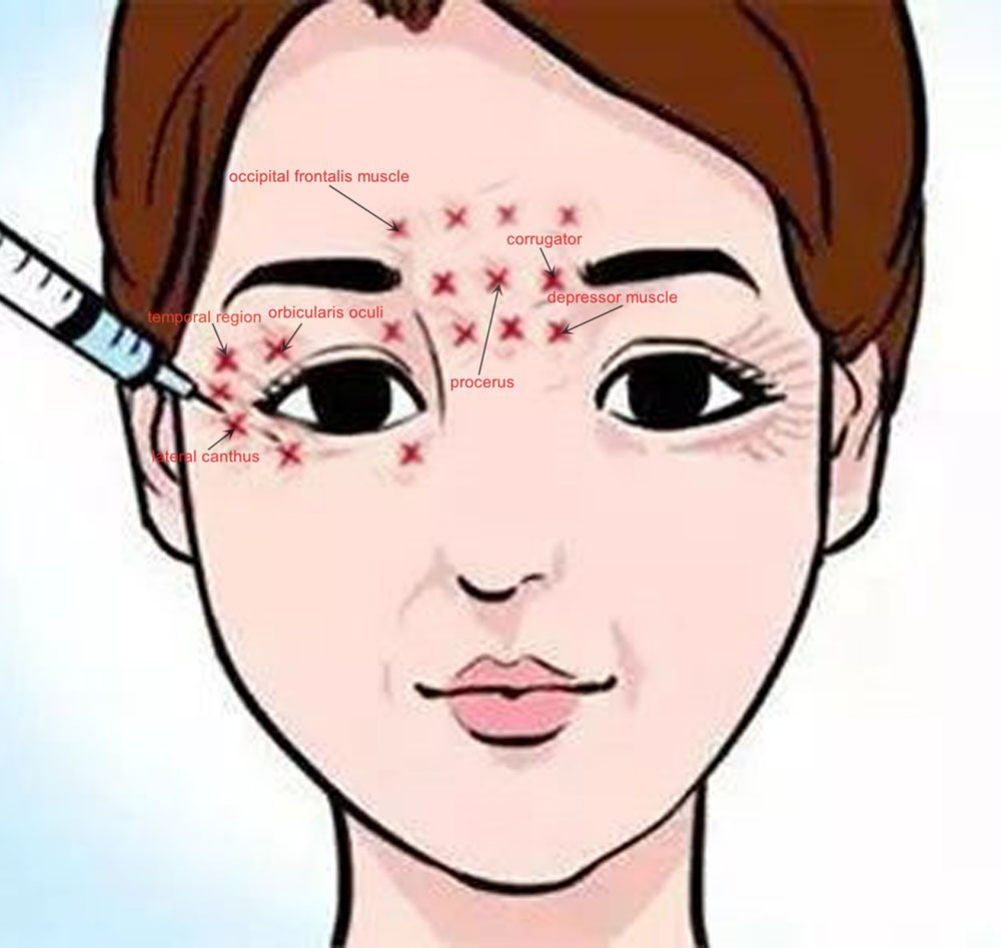
Schematic representation of BTX‐A injection sites (▲stands for the injection site, 10 points for the frowning muscle, depressor muscle, and occipital frontalis muscle, four points each for orbicularis oculi, three points each for lateral canthus, and the bilateral temporal region. A total of 24 sites and 3–4 units per site were applied for the BTX‐A.

PD patients manifesting neuropsychiatric symptoms received targeted intradermal infiltrations of BTX‐A (Hengli, 100U/vial, Lanzhou Biological Products Institute). Prior to administration, the toxin was reconstituted using sterile physiological saline (0.9% NaCl) under aseptic technique to achieve a working concentration of 40 units per milliliter. Injections were performed using BD PrecisionGlide single‐use sterile insulin syringes (1 mL capacity, 29G × ½ needle) following a standardized anatomical protocol. Neurotoxin was injected into predefined facial regions (bilateral glabella, forehead, orbicularis oculi muscle, bilateral lateral canthus, temporoparietal fascia) using a superficial subcutaneous technique. Each site received 3–4 U with total volume ≤1.5 mL/session (Figure 2). Individual dosages of BTX‐A were presented in Table S1.

### 2.9 Statistical Analyses

Quantitative variables conforming to normal distribution were expressed as mean ± standard deviation (SD) with intergroup comparisons via independent‐samples *t*‐tests, while nonnormally distributed continuous variables were reported as median [interquartile range (IQR)] and analyzed using Wilcoxon rank‐sum tests. Categorical variables appeared as frequencies (percentages) with between‐group differences evaluated through Pearson's chi‐square tests. Bivariate associations utilized Spearman's rank correlation coefficient. Treatment efficacy was modeled using multivariate logistic regression controlling for age, sex, disease duration, tobacco use, medication side effects, and baseline symptom severity. Statistical significance followed two‐tailed *p* < 0.05 criteria with appropriate multiplicity adjustments. All analyses employed R statistical software (version 4.1.1).

## 3 Results

### 3.1 Baseline Characteristics

The BTX‐A group included 36 males and 22 females, mean age of 61.04 ± 11.12 years, and with disease onset at 55.8 ± 9.3 years. A separate cohort of 39 PD patients with neuropsychiatric comorbidities received CH (21 males, 18 females; mean age 63.12 ± 10.96 years). Baseline demographics, comorbidities (hypertension, diabetes, coronary heart disease), and lifestyle factors showed no between‐group differences (all *p* > 0.05). Pretreatment psychiatric evaluations (HAMD, HAMA, SCL‐90, CMI) revealed comparable psychometric profiles between groups (all *p* > 0.05). CMI self‐assessment showed equivalent scores across domains: somatization (*p* = 0.42), anxiety (*p* = 0.82), depression (*p* = 0.54), sensitivity (*p* = 0.13), maladjustment (*p* = 0.67), anger (*p* = 0.51), and tension (*p* = 0.65), with total scores matching (*p* = 0.32). Notably, significant differences emerged in fatigue severity (*p* = 0.012), fatigue and habits (*p* = 0.02), and medical history and health status (*p* = 0.04), detailed in Table 1.

**TABLE 1:** Comparison of baseline data between the two groups.

Baseline data	BTX‐A group	CH group	*p*
	*N* = 58	*N* = 39	
Age mean (SD)	61.04 (11.12)	63.12 (10.96)	0.5
Gender			0.6
Male (*n* [%])	36 (62.1%)	21 (53.8%)	
Hypertension (*n* [%])	32 (55.2%)	20 (51.3%)	0.9
Diabetes (*n* [%])	21 (36.2%)	9 (23.1%)	0.3
Coronary heart disease (*n* [%])	16 (27.6%)	8 (20.5%)	0.6
Smoking history (*n* [%])–	18(31.0%)	8 (20.5%)	0.4
Alcohol consumption (*n* [%])	15(25.9%)	6 (15.4%)	0.3
UPDRS, mean (SD)	43.3(8.9)	42.5(8.6)	0.8
HAMA, mean (SD)	16 (4)	16 (5)	0.9
HAMD, mean (SD)	15 (6)	16 (6)	0.7
SCL‐90, Mean(SD)	178 (7)	177 (9)	0.5
CMI			
Somatization (median [IQR])	71 (24–112)	68 (23–108)	0.42
Fatigue and habits (median [IQR])	9 (4–15)	6 (2–14)	0.02
Medical history and health status (median [IQR])	12 (5–24)	10 (3–23)	0.04
Anxiety (median [IQR])	7 (2–9)	7 (3–9)	0.82
Depression (median [IQR])	5 (3–6)	4 (2–7)	0.54
Maladjustment (median [IQR])	9(4‐12)	8 (3–12)	0.67
Sensitivity (median [IQR])	3 (2–6)	4 (1–6)	0.13
Anger (median [IQR])	6 (2–9)	7 (2–9)	0.51
Tension (median [IQR])	7 (3–9)	8 (4–9)	0.65
Total scores (median [IQR])	130 (76–183)	127 (72–178)	0.32

### 3.2 Comparison of Various Factors of CMI Self‐Assessment Questionnaire in the BTX‐A Group Before and After BTX‐A Treatment

In the BTX‐A group, posttreatment assessments at 8 weeks revealed significant reductions in CMI subscale scores for somatization (*p* = 0.028), tension (*p* < 0.001), anxiety (*p* = 0.001), depression (*p* = 0.002), and sensitivity (*p* = 0.006) among PD patients with comorbid neuropsychiatric diagnoses. All mentioned improvements achieved statistical significance. By contrast, domains including fatigue and habits (*p* = 0.465), medical history and health status (*p* = 0.693), maladjustment (*p* = 0.574), and anger (*p* = 0.705) demonstrated nonsignificant changes relative to baseline measurements, with complete metric comparisons presented in Table 2.

**TABLE 2:** Comparison of various factors before and after treatment in the BTX‐A group.

Variables	Before treatment (*N* = 56)	After treatment (*N* = 56)	*p*
Somatization (median [IQR])	71 (24–112)	57 (21–104)	0.028
Fatigue and habits (median [IQR])	9 (4–15)	8 (4–14)	0.465
Medical history and health status (median [IQR])	12 (5–24)	13 (5–23)	0.693
Anxiety (median [IQR])	7 (2–9)	5 (2–9)	0.001
Depression (median [IQR])	5 (3–6)	3 (2–6)	0.002
Maladjustment (median [IQR])	9 (4–12)	8 (2–11)	0.574
Sensitivity (median [IQR])	3 (2–6)	3 (1–5)	0.006
Anger (median [IQR])	6 (2–9)	7 (4–9)	0.705
Tension (median [IQR])	7 (3–9)	5 (2–8)	<0.001
Total scores (median [IQR])	130 (76–183)	110 (66–162)	0.009

### 3.3 Comparison of the Efficacy Between the Two Groups

**FIGURE 3:**
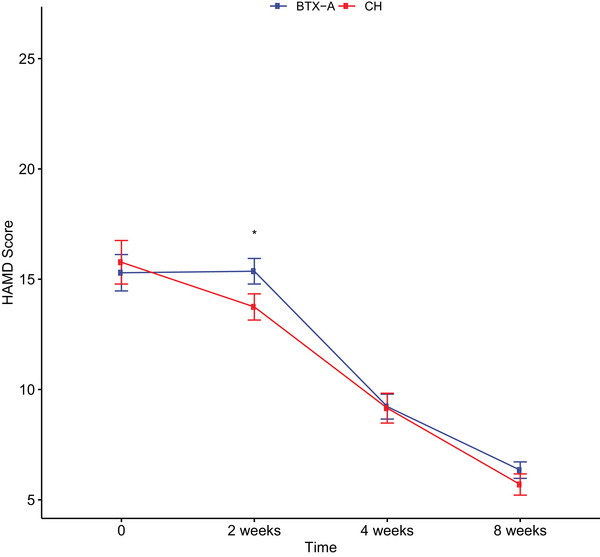
The difference between BTX‐A and CH groups at each time point was determined using the unpaired *t*‐test (*: *p* < 0.05). BTX‐A, botulinum toxin A; CH, citalopram hydrobromide; HAMD, Hamilton Depression Rating Scale.

**FIGURE 4:**
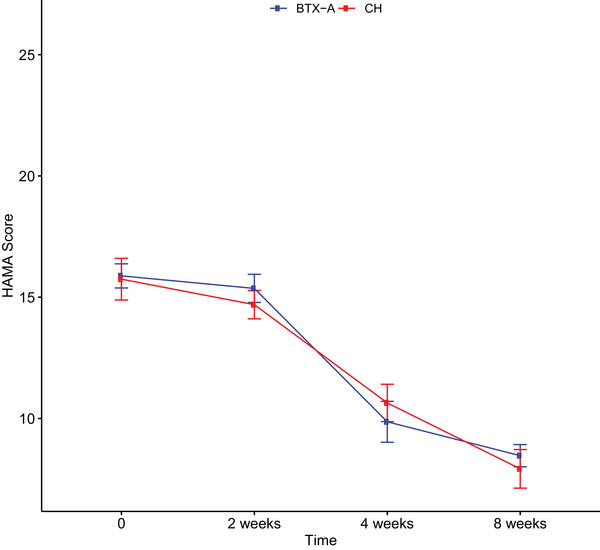
The difference between BTX‐A and CH groups at each time point was determined using the unpaired *t*‐test. BTX‐A, botulinum toxin A; CH, citalopram hydrobromide; HAMA, Hamilton Anxiety Rating Scale.

Eight‐week therapeutic courses with BTX‐A and CH demonstrated comparable efficacy profiles at all assessment intervals, though with distinct temporal response patterns (Figures 3 and 4). Temporal analysis revealed marked divergence at week 2, where BTX‐A recipients exhibited significantly lower HAMD scores, reflecting accelerated depressive symptom reduction. This time‐specific therapeutic advantage is graphically represented in Figure 3. At the 8‐week endpoint, treatment response rates reached 64.3% (36/56) in the BTX‐A group versus 73.7% (28/38) in the CH group. Treatment response categorization (improvement/stable/worsening) showed no statistically significant between‐group differences (*p* = 0.52/0.15/0.33), with detailed frequency distributions presented in Table 3.

**TABLE 3:** Comparison of the efficacy between the two groups at 8 weeks posttreatment.

Variables	BTX‐A group (*N* = 56)	Citalopram group (*N* = 38)	Unadjusted	Adjusteda	*p*
			OR (95% CI)	OR (95% CI)	Unadjusted/adjusted
Improvement, % (*n*)	64.3 (36)	73.7 (28)	0.65 (0.23–1.73)	0.59(0.21–1.69)	0.46/0.52
Deterioration, *%* (*n*)	0	7.9(3)	…	…	0.19/0.33
No change, % (*n*)	35.7 (20)	18.4 (7)	2.44 (0.85–7.77)	2.37 (0.82–7.62)	0.11/0.15

^a^Adjusted for factors such as age, gender, duration of the disease, smoking history, medication side effects, and Hoehn–Yahr staging.

### 3.4 The Correlation Between Hoehn–Yahr Grades and Neuropsychiatric Scales in Two Groups Prior to Treatment

Pre‐treatment evaluations revealed statistically significant correlations between HY staging and neuropsychiatric scales in PD patients with neuropsychiatric comorbidities, including: somatization (*r* = 0.31, *p* = 0.019), fatigue and habits (*r* = 0.12, *p* = 0.472), medical history and health status (*r* = ‐0.36, *p* = 0.033), sensitivity (*r* = 0.18, *p* = 0.391), depressive (*r* = 0.35, *p* = 0.022), anxiety (*r* = 0.51, *p* = 0.002), maladjustment (*r* = 0.28, *p* = 0.017), anger (*r* = 0.58, *p* < 0.001), tension (*r* = 0.49, *p* = 0.003), and total CMI scores (*r* = 0.52, *p* = 0.001), with complete correlation matrix presented in Table 4.

**TABLE 4:** Correlation analysis of Hoehn–Yahr staging with neuropsychiatric scales.

Variables	*r*	*p*
Somatization	0.31	0.019
Fatigue and habits	0.12	0.472
Medical history and health status	−0.36	0.033
Anxiety	0.51	0.002
Depression	0.35	0.022
Maladjustment	0.28	0.017
Sensitivity	0.18	0.391
Anger	0.58	<0.001
Tension	0.49	0.003
Total scores	0.52	0.001

## 4 Discussion

This study demonstrated that BTX‐A effectively alleviated tension, depression, anxiety, and sensitivity in PD patients, with efficacy comparable to CH. These findings align with prior studies and suggest potential mechanisms underlying BTX‐A's effects in PD.

CH, a selective serotonin reuptake inhibitor (SSRI), is frequently prescribed for the management of depression and anxiety disorders. The therapeutic effects of SSRIs, including CH, usually become evident within 2–4 weeks, although some patients may experience improvements sooner or later. CH specifically addresses depressive symptoms, such as low mood, loss of interest, and decreased energy, as well as anxiety symptoms, including excessive worry, restlessness, and irritability. On the other hand, the onset of therapeutic effects of BTX‐A may vary depending on the individual patient and the specific neuropsychiatric symptoms being treated. When comparing the therapeutic effects of CH and BTX‐A, it is crucial to recognize that they target distinct aspects of neuropsychiatric symptoms. CH primarily focuses on depressive and anxiety symptoms by modulating serotonin neurotransmission, whereas BTX‐A may have a more intricate mechanism of action, potentially involving the modulation of multiple neurotransmitter systems. Additionally, the specific symptoms that respond positively to each treatment may vary among individuals. In summary, both CH and BTX‐A demonstrate potential in treating neuropsychiatric symptoms associated with PD. However, they target different aspects of these symptoms and may exhibit varying onsets of therapeutic effects. Further research is warranted to elucidate the mechanisms of action of these treatments and to identify the specific symptoms that respond optimally to each therapy.

We observed significant reductions in somatization, tension, anxiety, depression, sensitivity, and overall scores in the BTX‐A group at the 8‐week follow‐up, as assessed by the CMI self‐assessment questionnaire. Notably, the improvements in these neuropsychiatric symptoms were comparable to those observed in the CH group, serving as a positive control. These results highlight the therapeutic potential of BTX‐A in managing neuropsychiatric manifestations in PD.

Consistent with observations by Lyu et al., our findings corroborate the antidepressant efficacy of BTX‐A in PD, demonstrating measurable reductions in depressive symptom severity across evaluated patients (Lyu et al. 2019). Our findings suggest that BTX‐A could play a role in mitigating tension and sensitivity; however, further studies are needed to validate these observations. Our study was not designed to definitively establish a causal link between BTX‐A and improvements in these specific neuropsychiatric symptoms, and thus, our interpretations must remain cautious and consistent with the scope and data of the study. Furthermore, our analysis revealed a significant positive association between the severity of neuropsychiatric manifestations and HY staging scores, suggesting that advancing PD stages are paralleled by proportional escalation in comorbid psychiatric burden.

Elucidating the neurobiological underpinnings of PD remains pivotal for advancing phenotypic characterization, diagnostic innovation, and therapeutic development. Beyond cardinal subcortical α‐synuclein deposits (Lewy bodies), PD pathogenesis involves widespread cortical involvement with diffuse Lewy pathology in prefrontal‐limbic‐neocortical circuits, striatal dopaminergic denervation, and dysregulation of nondopaminergic neurotransmission (cholinergic, serotonergic, noradrenergic) contributing to psychiatric comorbidities (Weintraub et al. 2022). Emerging evidence implicates neuroinflammatory processes, disrupted gut‐brain axis communication, cerebrovascular pathology, and polygenic susceptibility profiles in the manifestation of neuropsychiatric symptoms (Weintraub et al. 2022). While the precise pathophysiological mechanisms remain incompletely understood, recent investigations highlight multifactorial interactions between these systems. The therapeutic action of BTX‐A in managing depression and anxiety represents an evolving area of neuropsychiatric research. Though primarily recognized for its neuromuscular junction effects via SNARE protein inhibition, emerging data suggest central neuromodulatory properties mediating its psychiatric efficacy. Four putative mechanistic pathways warrant consideration: (1) *Somatosensory feedback disruption*: Injection‐mediated paralysis of facial mimetic muscles (e.g., corrugator supercilii) may attenuate afferent somatosensory signaling that reinforces negative affective states through bidirectional facial‐emotional feedback loops (Finzi and Rosenthal 2014; Finzi and Rosenthal 2016). (2) *Central neurotransmitter modulation*: Preclinical models demonstrate BTX‐A's capacity to regulate serotonergic tone and other neurotransmitter systems beyond peripheral sites of action, potentially normalizing limbic circuit dysfunction (Finzi and Rosenthal 2014; Li et al. 2019). (3) *Musculoskeletal‐affective axis*: By alleviating chronic myofascial tension—a bidirectional contributor to emotional distress—BTX‐A may interrupt pain‐psychopathology cycles through proprioceptive reprogramming (Zhang et al. 2017; Affatato et al. 2021). (4) *Psychoneuroimmunological interplay*: Contextual factors, including treatment expectancy, therapeutic alliance, and placebo response dynamics, likely modulate clinical outcomes, necessitating hybrid mechanistic models incorporating biological and psychosocial dimensions (Wollmer et al. 2021). Specifically, BTX‐A disrupts the facial feedback loop that reinforces depressive or anxious feelings by inhibiting facial muscles associated with negative emotional expressions. Furthermore, its widespread effects beyond the neuromuscular junction, particularly modulation of neurotransmitter systems, may contribute to its therapeutic benefits in neuropsychiatric symptoms. Moreover, despite discussing mechanisms such as facial feedback and neurotransmitter modulation, the precise neurobiological pathways through which BTX‐A exerts its effects in PD remain unclear. The relationship between these proposed mechanisms and the unique neurobiology of PD has not been clearly established. Future research is needed to further elucidate the neurobiological mechanisms underlying BTX‐A's therapeutic action in PD patients, potentially identifying novel targets and treatment strategies for neuropsychiatric symptoms.

Our findings uncover the potential of botulinum toxin in treating neuropsychiatric symptoms in PD and have significant implications for clinical practice. For patients with neuropsychiatric symptoms resistant to conventional therapies, botulinum toxin emerges as a novel treatment option. This could improve patients' quality of life and alleviate the burden on caregivers and healthcare institutions. Our research enables clinicians to accurately identify PD patient subgroups suitable for botulinum toxin treatment, particularly those with neuropsychiatric symptoms resistant to conventional therapies. This facilitates the development of personalized treatment regimens, enhancing therapeutic outcomes. Furthermore, the introduction of botulinum toxin offers PD patients a broader range of treatment options. Clinicians can tailor comprehensive treatment plans combining botulinum toxin with other therapeutic modalities (e.g., pharmacotherapy, physiotherapy) based on individual patient profiles, aiming for optimal therapeutic effects. Our study also suggests directions for future research. Specifically, further exploration of the synergistic effects of botulinum toxin combined with other medications, as well as the safety and efficacy of long‐term treatment, is necessary. This will contribute to the continuous refinement of treatment strategies for PD patients, ultimately enhancing their quality of life.

This study has several notable limitations. First, the sample size is relatively small, which may lack sufficient statistical power. Second, the experimental design, which is nonrandomized and double‐blind, may introduce bias in the research outcomes. Third, the follow‐up time is relatively short, potentially failing to capture long‐term treatment effects. Fourth, the clinical relevance of the observed improvements in neuropsychiatric scales remains uncertain, as there are no universally established thresholds for clinical significance for the scales used in this study. While we believe that the magnitude of change observed is likely to be clinically relevant based on our literature review and expert consultation, further research is needed to confirm this. Furthermore, baseline differences in factors such as fatigue and health status may have influenced treatment responses, highlighting the need for randomized controlled trials to reduce potential confounding. Therefore, to further validate our conclusions in future research, we plan to expand the sample size and conduct a randomized controlled trial.

Overall, our research provides an exploratory investigation into the potential use of BTX‐A in managing neuropsychiatric symptoms associated with PD. Due to the study design and the duration of follow‐up, it is important to acknowledge that additional data are required before definitive conclusions can be drawn. However, our preliminary findings indicate that BTX‐A may offer promise in this context, suggesting that further research is necessary to elucidate the neurobiological mechanisms underlying these symptoms. Such efforts are vital for advancing both the clinical management of PD and our comprehensive understanding of its pathology.

## Author Contributions

**Huimin Li**: data curation, formal analysis, writing – original draft. **Ming Wei**: conceptualization, project administration, methodology, investigation. **Xiaofeng Zhu**: methodology, project administration, investigation, data curation. **Qiang Tong**: formal analysis, visualization, funding acquisition, resources. **Xiu Yang**: writing—original draft, investigation, methodology, project administration. **Qiu Han**: conceptualization, writing—review & editing, writing—original draft, funding acquisition.

## Conflicts of Interest

The authors declare no conflicts of interest.

## Peer Review

The peer review history for this article is available at https://publons.com/publon/10.1002/brb3.70806.

## Supporting information




**Supporting Table**: brb370806‐sup‐0001‐TableS1.docx

## Data Availability

The data that support the findings of this study are available from the corresponding author upon reasonable request.
