# Supplementary material for: Efficacy of Facial Botulinum Toxin A Injections in Alleviating Neuropsychiatric Symptoms in Parkinson's Disease Patients: An Open‐Label, Nonrandomized Controlled Trial
Source: Brain Behav. 2025 Sep 8;15(9):e70806. doi: 10.1002/brb3.70806 (PMC12417628; doi:10.1002/brb3.70806)
Supplement: Supplementary file 1 — Supporting Table: brb370806‐sup‐0001‐TableS1.docx [file BRB3-15-e70806-s001.docx]

| group | patient ID | individual dosages (U) |
| --- | --- | --- |
| BTX-A | 1 | 72 |
| BTX-A | 2 | 88 |
| BTX-A | 3 | 94 |
| BTX-A | 4 | 73 |
| BTX-A | 5 | 88 |
| BTX-A | 6 | 93 |
| BTX-A | 7 | 82 |
| BTX-A | 8 | 94 |
| BTX-A | 9 | 85 |
| BTX-A | 10 | 91 |
| BTX-A | 11 | 75 |
| BTX-A | 12 | 94 |
| BTX-A | 13 | 76 |
| BTX-A | 14 | 88 |
| BTX-A | 15 | 90 |
| BTX-A | 16 | 81 |
| BTX-A | 17 | 74 |
| BTX-A | 18 | 76 |
| BTX-A | 19 | 85 |
| BTX-A | 20 | 73 |
| BTX-A | 21 | 83 |
| BTX-A | 22 | 79 |
| BTX-A | 23 | 85 |
| BTX-A | 24 | 83 |
| BTX-A | 25 | 78 |
| BTX-A | 26 | 89 |
| BTX-A | 27 | 92 |
| BTX-A | 28 | 77 |
| BTX-A | 29 | 91 |
| BTX-A | 30 | 96 |
| BTX-A | 31 | 73 |
| BTX-A | 32 | 87 |
| BTX-A | 33 | 80 |
| BTX-A | 34 | 91 |
| BTX-A | 35 | 93 |
| BTX-A | 36 | 94 |
| BTX-A | 37 | 87 |
| BTX-A | 38 | 72 |
| BTX-A | 39 | 93 |
| BTX-A | 40 | 85 |
| BTX-A | 41 | 90 |
| BTX-A | 42 | 81 |
| BTX-A | 43 | 82 |
| BTX-A | 44 | 77 |
| BTX-A | 45 | 85 |
| BTX-A | 46 | 84 |
| BTX-A | 47 | 86 |
| BTX-A | 48 | 78 |
| BTX-A | 49 | 82 |
| BTX-A | 50 | 85 |
| BTX-A | 51 | 94 |
| BTX-A | 52 | 83 |
| BTX-A | 53 | 82 |
| BTX-A | 54 | 88 |
| BTX-A | 55 | 94 |
| BTX-A | 56 | 85 |
| BTX-A | 57 | 91 |
| BTX-A | 58 | 78 |
